# Effects of Annealing on Electrochemical Properties of Solvothermally Synthesized Cu_2_SnS_3_ Anode Nanomaterials

**DOI:** 10.1186/s11671-021-03482-6

**Published:** 2021-01-28

**Authors:** Xiaoli Peng, Chong Wen, Qian Zhang, Hang Min, Yong Xiang, Xiaoran Hu, Xiaokun Zhang

**Affiliations:** School of Materials and Energy, University of Electronic Science and Technology of China, 2006 Xiyuan Ave, West High-Tech Zone, Chengdu, 611731 Sichuan China

**Keywords:** Cu_2_SnS_3_, Nanoparticles, Solvothermal method, Annealing, Anode

## Abstract

Cu_2_SnS_3_, as a modified material for high-capacity tin-based anodes, has great potential for lithium-ion battery applications. The solvothermal method is simple, convenient, cost-effective, and easy to scale up, and has thus been widely used for the preparation of nanocrystals. In this work, Cu_2_SnS_3_ nanoparticles were prepared by the solvothermal method. The effects of high-temperature annealing on the morphology, crystal structure, and electrochemical performance of a Cu_2_SnS_3_ nano-anode were studied. The experimental results indicate that high-temperature annealing improves the electrochemical performance of Cu_2_SnS_3_, resulting in higher initial coulombic efficiency and improved cycling and rate characteristics compared with those of the as-prepared sample.

## Introduction

Lithium-ion batteries are widely used in electric vehicles and portable electronic devices, but they require further improvements to parameters such as energy density, cycle life, power density, safety, and environmental compatibility [1–9]. Energy density is one of the most essential parameters as it determines the endurance mileage of an electric vehicle. Conventional lithium-ion batteries are limited by the specific capacity of the commercial graphite anode (LiC_6_, 372 mAh g^−1^). Therefore, it is critical to research anodes with high specific capacities to improve the energy density of lithium-ion batteries. In this regard, Sn-based anode materials have attracted attention due to their high specific capacities (Li_4.4_Sn, ~ 993 mAh g^–1^) [10–14]. However, the materials exhibit poor conductivities and large volume expansions (up to 300%) [15–17], causing low rates and poor cycling stabilities. Various strategies have been explored to improve the electrochemical properties of Sn-based anode materials [18–21]. Inert and non-inert elements introduced to form Sn-based composite materials can function as a buffer matrix for volume expansion, thereby improving the structural and cycling stability of the material. The inert elements that are often used to form Sn-based composites include Ni, Co, Mn, and Cu [22–27], and the non-inert elements include Sb, Ge, and the like [28–30]. Nanomerization of electrode materials can not only effectively inhibit volume changes during battery cycling and release the internal stress of the material, thereby improving its structural stability, but can also increase the specific surface area of the electrode, which promotes rapid reactions at the electrode interface. Furthermore, nanomerization can significantly reduce the diffusion distance of lithium ions in the active material, which reduces the polarization phenomenon of the electrode and improves the rate performance of the lithium-ion battery. Cai and Li reported that porous SnS nanorods/carbon hybrid nanostructure exhibited improved reversible capacity and cycling performance [31]. 3D hollow CoS@PCP/CNTs composites constructed by porous carbon/carbon nanotube-stabilized cobalt sulfide nanoparticles exhibited an ultrahigh reversible capacity of about 1668 mAh g^−1^ in the 100 cycles and an exceptional high-rate capability (1038, 979, 858, and 752 mAh g^−1^ at current densities of 1, 2, 5, and 10 A g^−1^, respectively) [32]. Cu_2_SnS_3_, as a modified material for high-capacity Sn-based anodes created by the introduction of inert Cu to form an alloy, has great potential for lithium-ion battery applications [17, 33–35]. Cu_2_SnS_3_ (CTS) nanostructure materials were successfully prepared via a facile solvothermal method for sodium-ion battery. The annealed CTS electrodes exhibit high initial reversible capacity 447.7 mAh g^−1^ and good capacity retention 200.6 mAh g^−1^ after 50 cycles at a current density of 100 mA g^−1^[36]. Fu and Li used a facile hydrothermal method to prepare Cu_2_SnS_3_/reduced graphene oxide (CTS/RGO) composite for sodium-ion batteries. CTS/RGO exhibits a high reversible capacity of 566.8 mA h g^−1^ and maintains a specific capacity of 339.8 mA h g^−1^ after 100 cycles at a constant current density of 100 mA g^−1^[37]. High-temperature sintered sulfides have been widely used to improve electrochemical performance. The effects of the high-temperature annealing process on the electrochemical performance for lithium-ion batteries of Cu_2_SnS_3_ were investigated in this paper.

The solvothermal method is simple, convenient, cost-effective, and easily scalable, and has thus been widely used for the preparation of nanocrystals. In this work, Cu_2_SnS_3_ nanoparticles for lithium-ion batteries were prepared herein by the solvothermal method. Furthermore, the effects of high-temperature annealing on the morphology, crystal structure, and electrochemical performance of Cu_2_SnS_3_ nano-anodes were studied.

## Experimental Section

### Materials Preparation

CuCl_2_⋅2H_2_O (99.9%), SnSO_4_ (99.9%), elemental sulfur powder (99.9%), and anhydrous ethylenediamine (99%) were purchased from Chengdu Kelong Chemical Co.

For the synthesis of Cu_2_SnS_3_ nanoparticles, CuCl_2_·2H_2_O (0.682 g, 4 mmol) and SnSO_4_ (0.473 g, 2.2 mmol) were first dissolved in deionized water with magnetic stirring for 20 min. The resulting mixture was loaded into an autoclave with a 25-ml Teflon container preloaded with a solution of sulfur powder (0.290 g, 9 mmol) suspended in anhydrous ethylenediamine. The airtight autoclave was transferred into an oven and heated from room temperature to 200 °C, held for 24 h, then naturally cooled to room temperature. The resulting precipitate was washed with deionized water several times and collected by centrifugation at 6000 rpm for 3 min to remove by-products. Then, the resulting precipitate was vacuum-dried at 80 °C for 10 h prior to use. The Cu_2_SnS_3_ nanoparticles were annealed at 540 °C for 40 min in a tubular furnace that was vacuumed and purged with nitrogen gas at a flow rate of 50–80 ml min^–1^ under ambient pressure.

### Materials Characterization

X-ray powder diffraction (XRD) data were acquired using a Bruker D8 ADVANCE with a Cu-Kα (λ = 1.5418 Å) radiation source. Scanning electron microscopy (SEM) (Hitachi S3400) and transmission electron microscopy (TEM) (Tecnai G2-F30-S-TWIN, FEI) were used to investigate the microstructures of the Cu_2_SnS_3_ nanoparticles. The composition of the sample was analyzed using energy-dispersive X-ray (EDX) spectroscopy. X-ray photoelectron spectra (XPS) of the Cu_2_SnS_3_ nanoparticles were obtained using an X-ray photoelectron spectrometer (ESCALAB 250Xi, Thermo Scientific).

### Battery Assembly and Electrochemical Measurements

The electrochemical performance of the Cu_2_SnS_3_ nanoparticles was tested with CR2032-type coin cells using Li metal as the counter electrode. The anode was composed of 80wt% active material, 10wt% super P, and 10wt% PVDF. The electrolyte was 1 M LiPF_6_ (EC:EMC:DEC = 4:2:4, vol%). The Cu_2_SnS_3_ electrodes were punched into circles with 12 mm diameter. The mass loading of Cu_2_SnS_3_ active material is 2.65 mg/cm^2^. The thickness of the casted Cu_2_SnS_3_ anode is ~ 30 μm, which is determined by micrometer. Cyclic voltammetry (CV) was performed at 0.1 mV s^−1^ from 2.0 to 0.0 V using a potentiostat (VersaSTAT3F, Princeton Applied Research). Cycling and rate tests were conducted in an automatic galvanostatic charge–discharge unit (CT-4800 battery testing system, Neware) between 0.05 and 2.0 V at room temperature. Electrochemical impedance spectroscopy was carried out using a potentiostat (VersaSTAT3F, Princeton Applied Research) within a frequency range from 100 kHz to 0.1 Hz.

## Results and Discussion

**Fig. 1:**
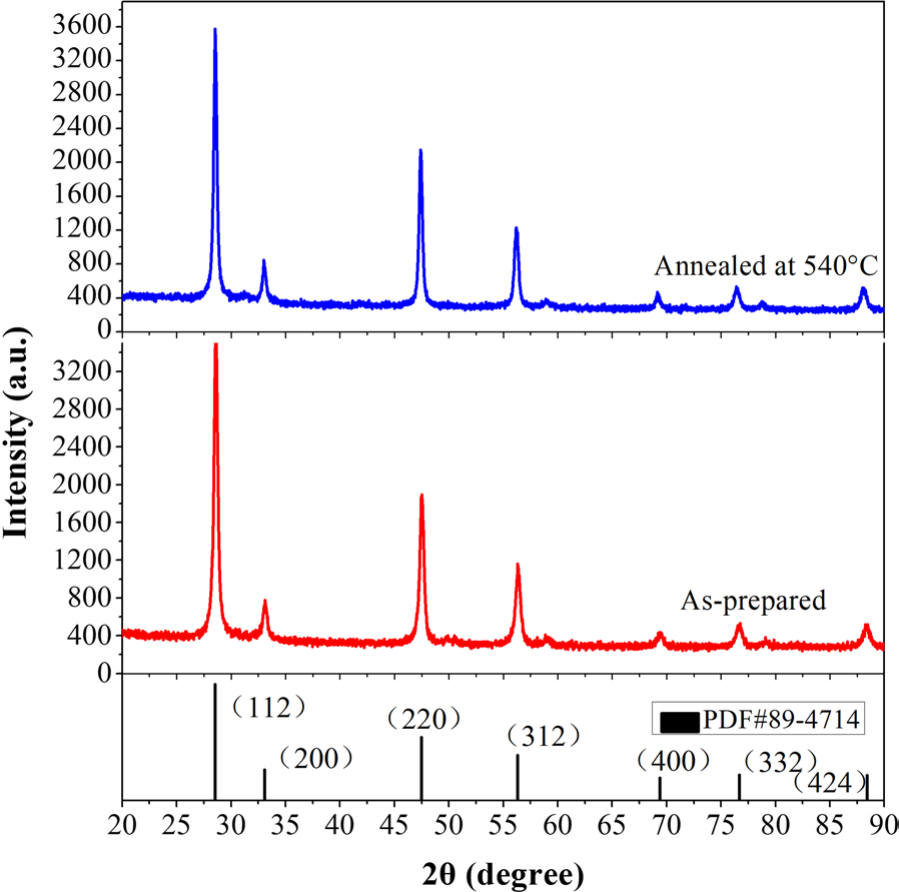
XRD spectra of Cu_2_SnS_3_ nanoparticles

Figure 1 shows the XRD patterns of the Cu_2_SnS_3_ nanoparticles. The diffraction peaks for both samples at 28.61°, 33.13°, 47.5°, 56.31°, 69.42°, 76.65°, and 88.44° could be assigned to the (112), (200), (220), (312), (400), (332), and (424) planes, respectively. The main diffraction peaks of the as-prepared and annealed Cu_2_SnS_3_ are well matched with those of tetragonal Cu_2_SnS_3_ (JCPDS 89-4714) [38, 39, 42], and no secondary phases were detected, confirming that the products were all phase pure with preferential growth along the (112) plane. After annealing at 540 °C, the relative intensity of each major diffraction peak in the XRD pattern increased, and the full-width at half-maximum (FWHM) of the (112) diffraction peak decreased from 0.4 to 0.35, indicating that the annealing process enhanced the crystallinity of the material [40, 41].

**Fig. 2:**
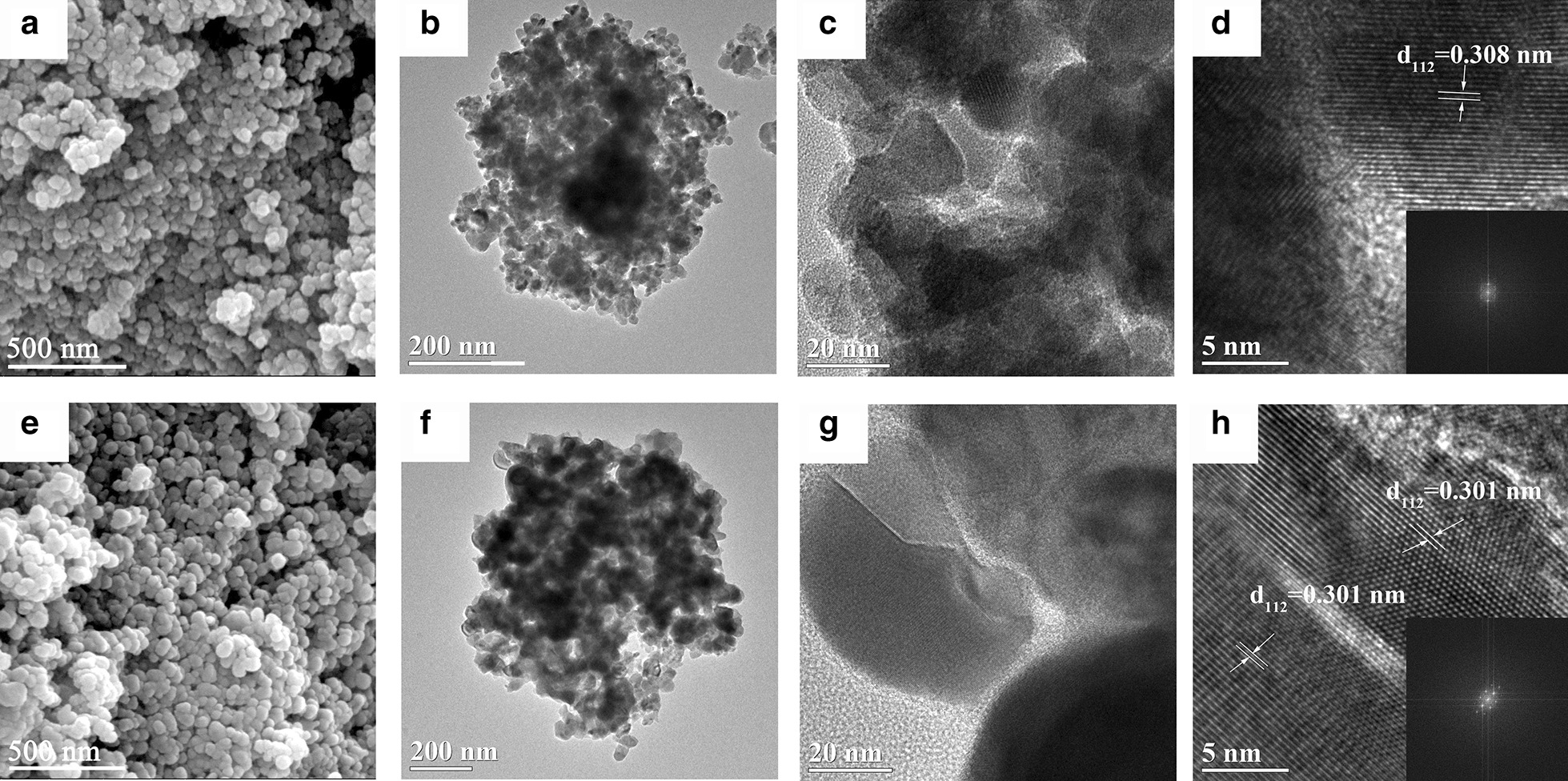
**a** SEM, **b**, **c** TEM, and **d** HRTEM images of as-prepared Cu_2_SnS_3_ nanoparticles; **e** SEM, **f**, **g** TEM, and **h** HRTEM images of annealed Cu_2_SnS_3_ nanoparticles

As shown in the SEM images in Fig. 2a, e, the as-prepared and annealed Cu_2_SnS_3_ were present in the shape of spherical nanoparticles, which aggregate to form irregular ball-like agglomerates. The micron-shaped irregular spherical clusters formed by the primary nanoparticles are advantageous as they increase the compaction density of the anode and thereby increase the capacity of the battery. In order to further analyze the particle morphology and size, as well as the detailed internal crystal structural of Cu_2_SnS_3_, the Cu_2_SnS_3_ nanoparticles were further observed via TEM and HRTEM. As shown in Fig. 2c, g, the sizes of the as-prepared and annealed Cu_2_SnS_3_ particles were approximately 25 and 41 nm, respectively, and both materials further aggregated into 1-µm spherical particles as shown in Fig. 2b, f. In the HRTEM images shown in Fig. 2d, h, the lattice fringes are clearly observable, where the fringes of the annealed Cu_2_SnS_3_ nanoparticles (Fig. 2h) are more regular than those of the as-prepared sample. This further proved that the crystallization of the Cu_2_SnS_3_ nanoparticles was enhanced by annealing at 540 °C. Fast Fourier transform (FFT) of the high-resolution TEM of Cu_2_SnS_3_ is shown in frame Fig. 2d, h. The diffraction patterns of the materials are clearly shown in the FFT. The lattice spacing of 0.301 nm is close to the inter-planar distance of the (112) plane of Cu_2_SnS_3_. Thus, the HRTEM results are in good agreement with the XRD results (Fig. 1).

**Fig. 3:**
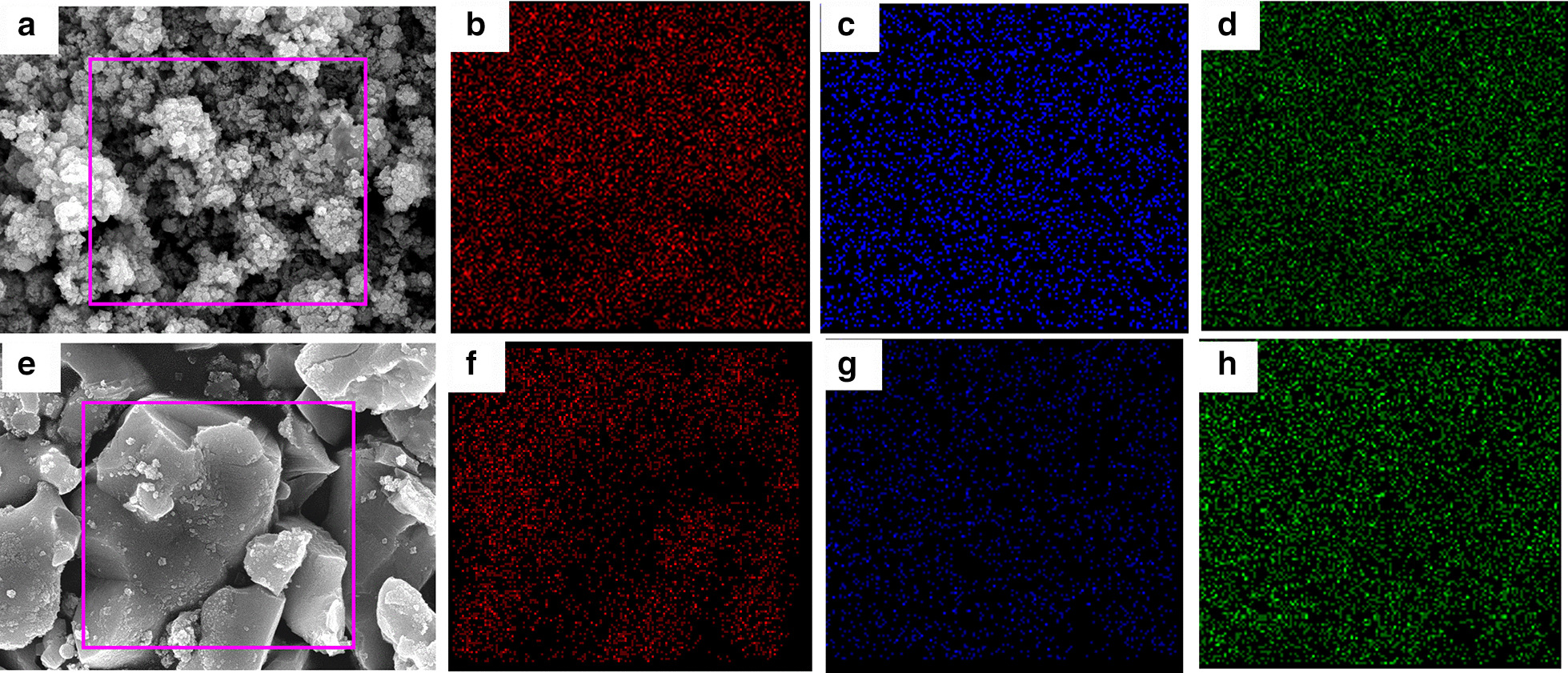
EDX elemental mapping of **b** Cu, **c** Sn and **d** S of the as-prepared CTS; **f** Cu, **g** Sn and **h** S of the annealed CTS

In order to investigate the distribution of Cu_2_SnS_3_, energy-dispersive X-ray (EDX) mapping was carried out. Elemental mapping images show the clear profiles of Cu, Sn, and S elements in the composite (Fig. 3). The results indicate the uniform distribution of Cu, Sn, and S elements in the CTS. EDX data verify that the element ratios of Cu:Sn:S for as-prepared Cu_2_SnS_3_ are 2:0.87:2.25. However, the element ratios of Cu:Sn:S = 2:1.006:2.89 for annealed Cu_2_SnS_3_ are approximately consistent with the stoichiometry.

**Fig. 4:**
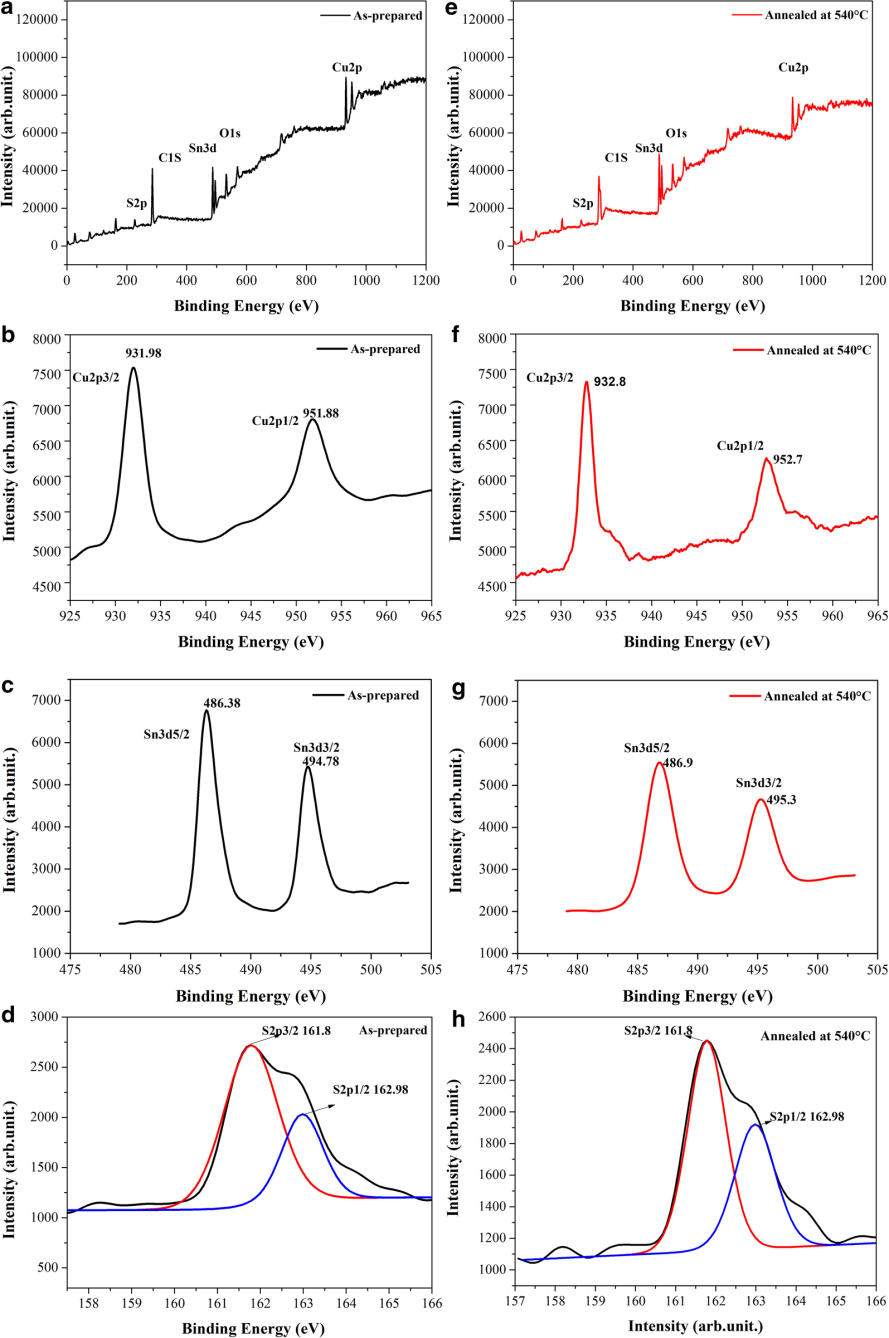
XPS profiles of as-prepared Cu_2_SnS_3_ nanoparticles: **a** typical survey spectrum, **b** Cu2*p* core level, **c** Sn3*d* core level, and **d** S2*p* core level. XPS spectra of annealed Cu_2_SnS_3_ nanocrystals: **e** typical survey spectrum, **f** Cu2*p* core level, **g** Sn3*d* core level, and **h** S2*p* core level

The valence states and composition of the Cu_2_SnS_3_ nanoparticles were further determined by XPS. Figure 4a, e shows the full XPS spectra of the as-prepared and annealed Cu_2_SnS_3_ nanoparticles, respectively. Cu, Sn, and S elements, as well as C (C1*s*, 285.08 eV) and O (O1*s*, 533.08 eV), were observed, and no other impurity elements were detected; the C and O impurity peaks may be due to environmental contamination [39, 42–47]. Figure 4b shows the Cu2*p* core-level spectrum of the as-prepared Cu_2_SnS_3_ nanoparticles. The binding energies for Cu2*p*3/2 and Cu2*p*1/2 occurred at 931.9 and 951.9 eV, respectively, which are consistent with the values of Cu^+^ reported in the literature [45, 47]; in contrast, the Cu^2+^ peak at 942 eV was not observed [48]. The binding energies of Sn3*d*5/2 and Sn3*d*3/2 for the as-prepared Cu_2_SnS_3_ nanoparticles occurred at 486.4 and 494.8 eV, respectively, corresponding to the Sn^4+^ values reported in the literature [45–47]. Figure 4f shows the Cu2*p* core-level spectrum of the annealed Cu_2_SnS_3_ nanoparticles; the binding energies for Cu2*p*3/2 and Cu2*p*1/2 occurred at 932.8 and 952.7 eV, respectively, which are also consistent with the values reported in the literature [39, 46]. The binding energies of Sn3*d*5/2 and Sn3*d*3/2 for the annealed Cu_2_SnS_3_ nanoparticles occurred at 486.9 and 495.3 eV (Fig. 4g), respectively, confirming the presence of Sn^4+^ [38, 39]. The binding energies of S2*p*3/2 and S2*p*1/2 for both the as-prepared and annealed Cu_2_SnS_3_ nanocrystals were 161.8 and 162.98 eV, respectively, indicating the presence of S. These values are consistent with those reported in the literature, which provides evidence for the existence of S^2−^ [43–47]. Consequently, the XPS results suggest that the Cu, Sn, and S elements in the as-prepared and annealed Cu_2_SnS_3_ nanoparticles are present in the ionic states of Cu^+^, Sn^4+^, and S^2−^, respectively. The annealing process enhances the crystallinity of Cu_2_SnS_3_ particles and increases the particle size. This phenomenon may cause changes in the electron cloud around the cations and increase the binding energy of Cu and Sn.

**Fig. 5:**
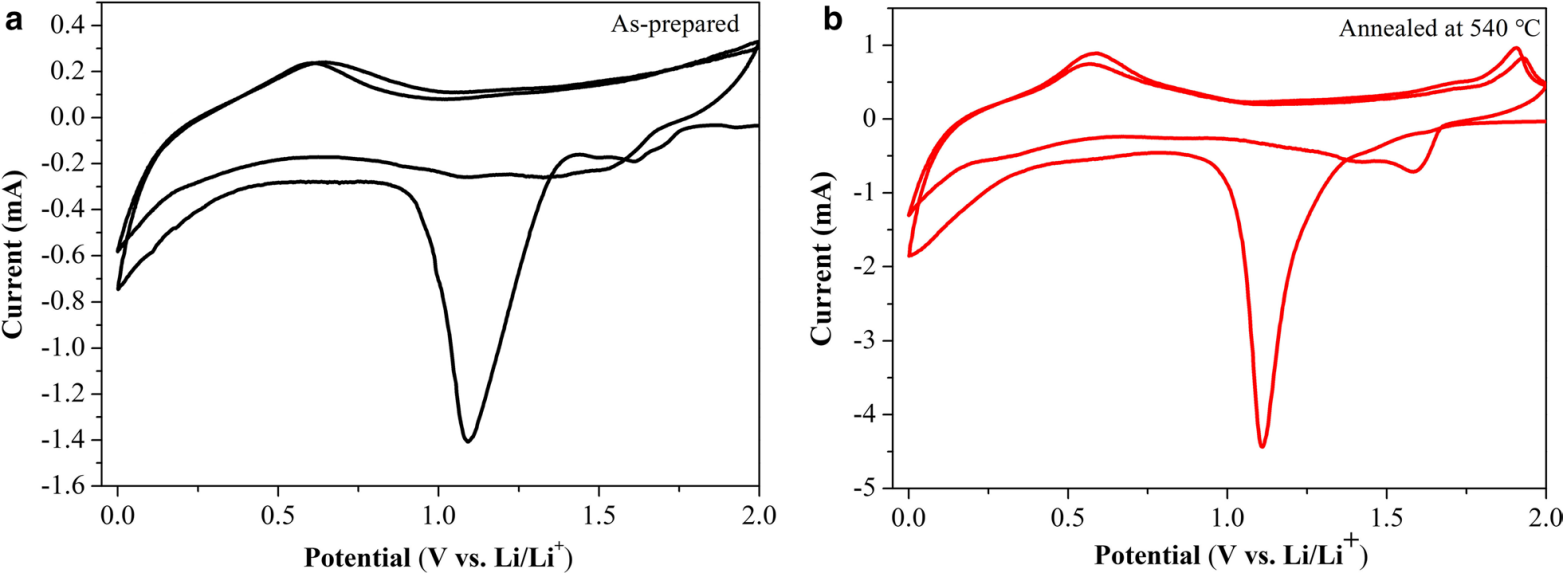
CV plots of initial two cycles scanned between 0 and 2 V at a rate of 0.1 mV s^–1^: **a** as-prepared and **b** annealed at 540 °C

Figure 5 shows the obtained CV plots for Cu_2_SnS_3_ from the initial two cycles scanned from 2 to 0 V at a rate of 0.1 mV s^–1^. Based on Fig. 5a, during the first lithium intercalation process, the as-prepared Cu_2_SnS_3_ nanoparticles showed large reduction peaks at approximately 1.09, the valence states of Cu^+^, Sn^2+^changing to Cu, Sn. The large reduction peaks at approximately 1.62 V are the reduction peak of H_2_O, and the current peak gradually disappeared in the second cycle. In the delithiation process, the oxidation current peak appeared at 0.62 V corresponding to formula (1), Sn with Li ions to form Li_x_Sn, and in the second cycle, the current peak remained basically unchanged. As shown in Fig. 5b, during the first lithium intercalation process, the Cu_2_SnS_3_ nanoparticles annealed at 540 °C showed a large reduction peak near 1.1 V corresponding to formula (1) [33], where Cu_2_SnS_3_ was reduced to Cu and Sn, and the current peak gradually increased to 1.59 V during the second cycle. The hump below 0.5 V corresponds to the conversion of Sn to Li_*x*_Sn according to formula (2) [33]. In the delithiation process, oxidation current peaks occurred at 0.59 and 1.94 V, and as the number of cycles increased, the current peak remained basically unchanged. The anodic peak at approximately 0.59 V is attributed to the Li_*x*_Sn alloy forming Sn, and the peak at 1.94 V corresponds to the inverse reaction of formula (1) [33]. The irreversible capacity loss, which arises from the formation of partially amorphous Li_2_S irreversibly consuming Li, causes the changes in potential and peak current intensity between the first and second cycles [17]. By comparison, the annealing treatment improved the cycling reversibility of the Cu_2_SnS_3_ nano-anodes.

1 $${\text{Cu}}_{2} {\text{SnS}}_{3} + 6{\text{Li}}^{ + } + 6{\text{e}}^{ - } \leftrightarrow 2{\text{Cu}} + {\text{Sn}} + 3{\text{Li}}_{2} {\text{S}}$$

2 $${\text{Sn}} + x{\text{Li}}^{ + } + x{\text{e}}^{ - } \leftrightarrow 4{\text{Li}}_{x} {\text{Sn}}\quad \left( {0 \, \le x \le \, 4.4} \right)$$

**Fig. 6:**
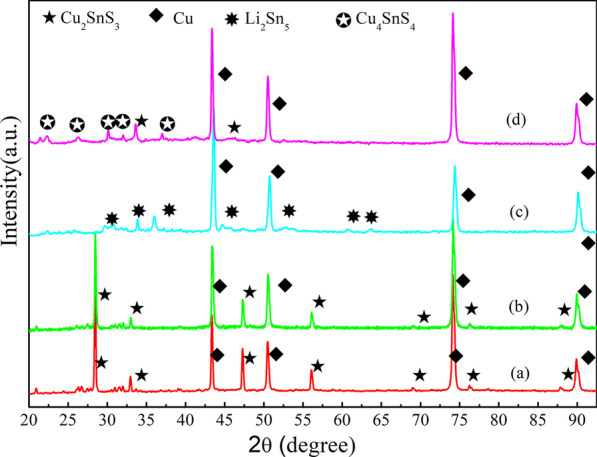
Ex situ XRD patterns of the electrode; **a** as-prepared; **b** first discharged to 1.5 V; **c** first discharged to 0.05 V; **d** second charged to 2 V

In order to fully understand the charge–discharge process, the ex situ XRD on Cu_2_SnS_3_ was performed on the electrodes after discharged and charged at the selected voltages as shown in Fig. 6. The coin cells were discharged/charged to different voltages and then equilibrated for 6 h. The cells were then disassembled inside the glovebox, and the Cu_2_SnS_3_ composite electrodes were washed with solvent DEC to remove the electrolyte. After the first discharge to 1.5 V, the crystal structure is not destroyed at 1.5 V as can be seen from Fig. 6b, and the main diffraction peaks of Cu_2_SnS_3_ composite electrodes are well matched with those of tetragonal Cu_2_SnS_3_ (JCPDS 89-4714), and no secondary phases were detected. After the first discharge to 0.05 V, Fig. 6c, the reflection peaks of Cu_2_SnS_3_ completely disappeared and Cu peaks became stronger and peaks of Li_2_Sn_5_ appeared. Such phenomena can be explained by the CTS decomposing into Cu and Sn nanoparticles, the Sn-forming Li_x_Sn. A reversible process happened when charged to 2 V, leading to recovery of the CTS phases, and formed Cu_4_SnS_4_.

**Fig. 7:**
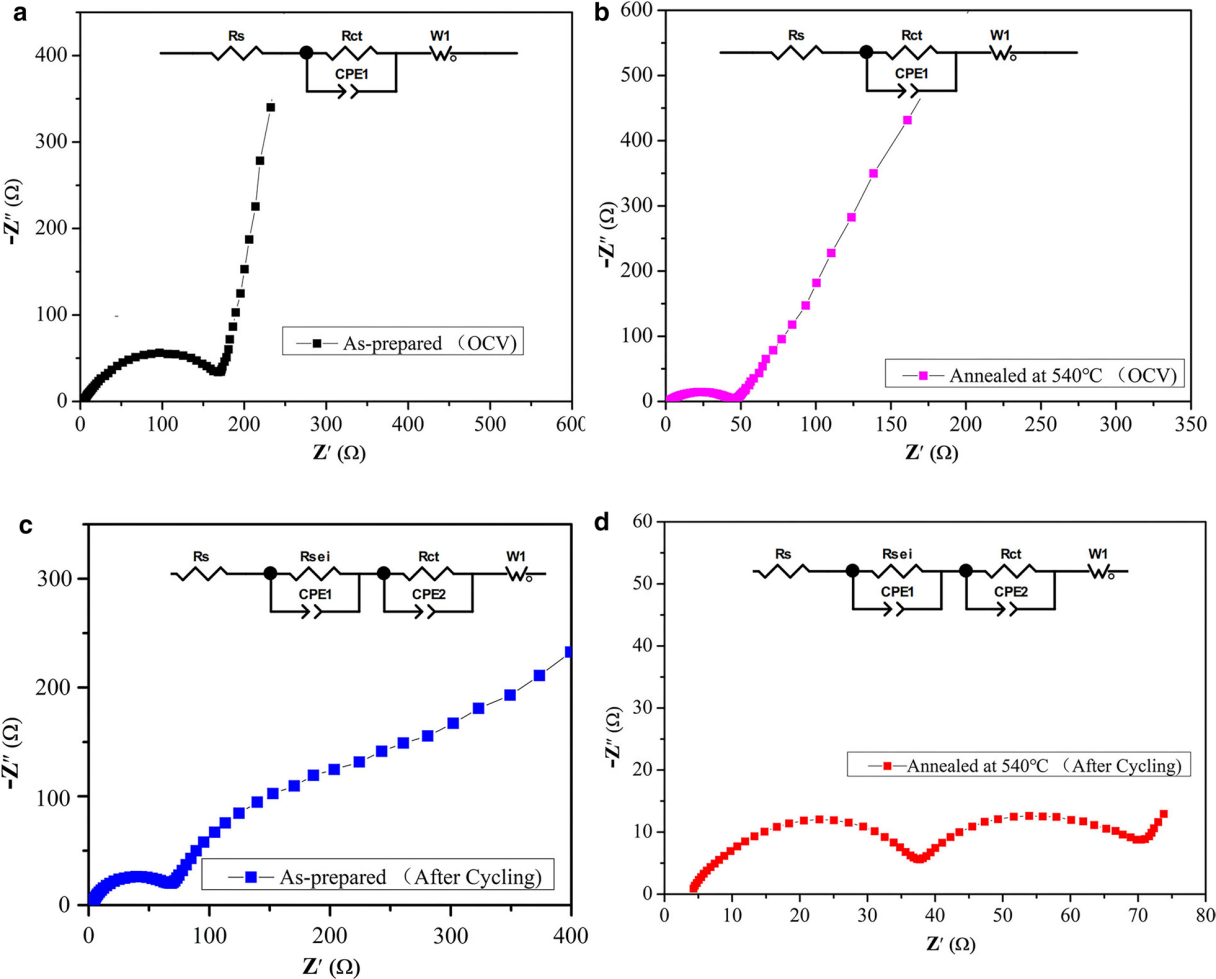
Nyquist plots of as-prepared and annealed Cu_2_SnS_3_ electrodes at OCV, after 2 cycles at 100 mA g^−1^ (0–2 V)

Figure 7 shows the Nyquist plots of the Cu_2_SnS_3_ electrodes at OCV, after 2 cycles at 100 mA g^−1^ (0–2 V). In the Nyquist plots of the Cu_2_SnS_3_ electrodes at OCV (Fig. 7a,b), a semicircle in the high-frequency region is attributed to the charge transfer resistance R_ct_ and a straight sloping line in the low-frequency region is ascribed to Li^+^ diffusion process in the bulk Zw [18, 49]. The R_ct_ of the annealed Cu_2_SnS_3_ electrode is less than that of the as-prepared electrode. In the Nyquist plots of the Cu_2_SnS_3_ electrodes after 2 cycles (Fig. 7c, d), a semicircle in the high-frequency region is attributed to the resistance of Li^+^ diffusion through the surface film R_sei_, a semicircle in middle-frequency region is assigned to the charge transfer resistance R_ct_, a straight sloping line in the low-frequency region is ascribed to Li^+^ diffusion process in the bulk Zw. The experimental datas are simulated by ZView software, which are obtained according to the equivalent circuit, and the values are listed in Table 1. We can find that there is no significance difference of the Ohm resistance (R_s_) between the as-prepared and annealed Cu_2_SnS_3_. However, the R_sei_ and R_ct_ values of annealed Cu_2_SnS_3_ are much smaller than that of as-prepared Cu_2_SnS_3_. Especially, the R_ct_ of the pristine sample is 162.4Ω at OCV and drastically increases to 206.6Ω after 2 cycles. In contrast, the R_ct_ of the annealed sample is 39.7Ω at OCV and drastically decreases to 25.9Ω after 2 cycles. The annealing process can suppress the SEI layer and charge transfer resistance, thereby facilitating charge transfer and ion conduction. As a consequence, the electrochemical performance of annealed Cu_2_SnS_3 _is improved.

**Table 1:** Fitting values of the electrochemical impedance profiles of the as-prepared and annealed Cu_2_SnS_3_

Sample	R_S_ (Ω)	R_ct_ (Ω)	R_sei_ (Ω)
As-prepared (OCV)	4	162.4	
Annealed at 540℃ (OCV)	2.4	39.7	
As-prepared (after 2 cycles)	3	206.6	61.2
Annealed at 540℃ (after 2 cycles)	3.9	25.9	35.3

**Fig. 8:**
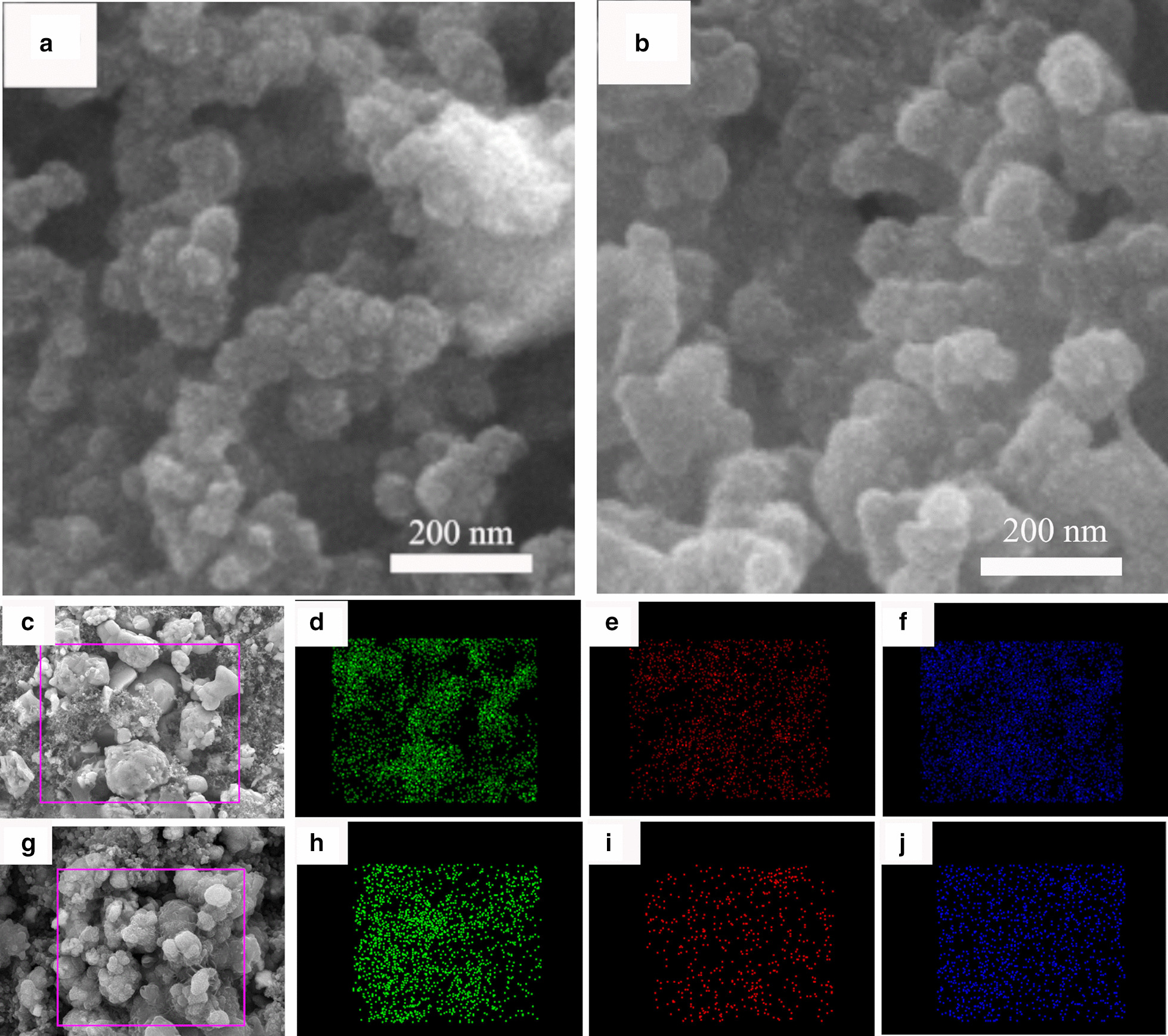
SEM images of annealed Cu_2_SnS_3_ nanoparticles: **a** at OCV, **b** after 5 cycles. EDX elemental mapping of **d** Cu, **e** Sn and **f** S of the annealed CTS electrode at OCV, **h** Cu, **i** Sn and **j** S of the annealed CTS electrode after 5 cycles

As shown in the SEM images in Fig. 8a, b, after 5 cycles, the shape of annealed Cu_2_SnS_3_ was not changed, showing spherical nanoparticles which aggregate to form irregular ball-like agglomerates. Elemental mapping images show the clear profiles of Cu, Sn, and S elements in the composite (Fig. 8d–f, h–j). The results indicate the uniform distribution of Cu, Sn, and S elements in the annealed CTS electrode after 5 cycles.

**Fig. 9:**
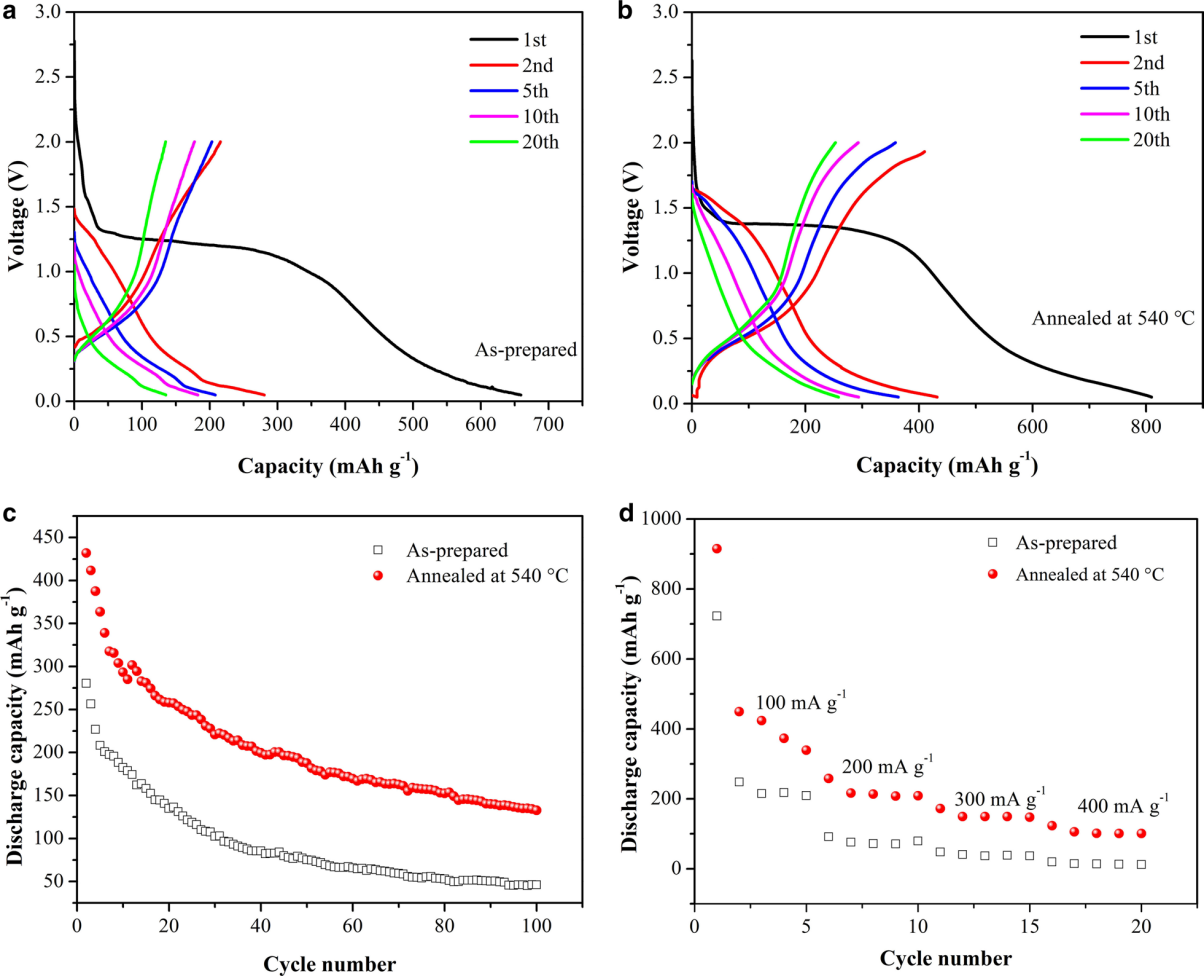
Charge–discharge curves of Cu_2_SnS_3_ electrodes (100 mA g^−1^): **a** as-prepared and **b** annealed at 540 °C and **c** cycling performance (100 mA g^−1^); **d** rate capability of as-prepared and annealed Cu_2_SnS_3_ electrodes at varying current densities (100 to 400 mA g^−1^)

Galvanostatic charge–discharge curves of the as-prepared and annealed Cu_2_SnS_3_ electrodes (Fig. 9a, b) were recorded at 100 mA g^–1^ over a potential range from 2 to 0 V (vs. Li/Li^+^). The initial discharge capacities of 654 and 809 mA g^–1^ correspond to initial coulombic efficiencies of 42% and 53%, respectively. The loss of irreversible capacity may be attributed to the formation of an SEI film and Li_2_S. Obviously, the annealing treatment improved the discharge capacity and initial coulombic efficiency of the Cu_2_SnS_3_ electrode.

The cycling performance of the as-prepared and annealed Cu_2_SnS_3_ electrodes to 100 cycles at a constant 100 mA g^–1^ is shown in Fig. 9c. It is clear that the discharge specific capacity of the annealed Cu_2_SnS_3_ electrode is overall superior to that of the as-prepared electrode. The capacity of annealed Cu_2_SnS_3_ electrode after 50 cycles is 187.7 mAh g^−1^, which is higher than that as-prepared electrode (75.2 mAh g^−1^). The capacity retention of annealed composites is almost equal to or better than the reports of hollow microspheres Cu_2_SnS_3_ and Cu_2_SnS_3_ nanosheets [34, 50]. But the capacity retention is much lower than Cu_2_SnS_3_/RGO composite (561 mAh g^−1^ after 100 cycles) [33]. The annealing process has been proved to improve the cycle performance of Cu_2_SnS_3_, but in the follow-up research, it is necessary to combine with other modification methods to further improve its performance.

As shown in Fig. 9d, the as-prepared Cu_2_SnS_3_ cells exhibited maximum discharge capacities of approximately 222, 78, 40, and 14 mAh g^–1^ at 100, 200, 300, and 400 mA g^–1^ discharge rates, respectively, with a discharge capacity retention ratio of only 6%. In contrast, the discharge specific capacities of the annealed Cu_2_SnS_3_ batteries were 396, 221, 153, and 106 mAh g^–1^ at 100, 200, 300, and 400 mA g^–1^ discharge rates, respectively, with a discharge capacity retention ratio of 26.8%. Obviously, the annealing treatment increased the crystallinity of Cu_2_SnS_3_ and led to a more stable crystal structure. Cu_2_SnS_3_ is polycrystalline and thus contains many grain boundaries. During the charge–discharge process, the mechanical stress produced by volume expansion of the internal particles can be buffered by the sliding of the grain boundaries, thus reducing the fracturing and pulverization of the material and stabilizing the electrode structure. This is beneficial to improving the cycling stability and rate characteristics of the nano-Cu_2_SnS_3_ anode.

## Conclusion

Cu_2_SnS_3_, as a modified material for high-capacity Sn-based anodes created by the introduction of inert Cu to form an alloy, has great potential for lithium-ion battery applications. Annealing at 540 °C increases the crystallinity of the Cu_2_SnS_3_ nanoparticles and leads to a more stable crystal structure. The high-temperature annealing treatment improves the electrochemical performance of Cu_2_SnS_3_, resulting in a higher initial coulombic efficiency and improved cycle and rate characteristics compared with those of the as-prepared sample.

## Data Availability

The authors declare that materials and data are promptly available to readers without undue qualifications in material transfer agreements. All data generated or analyzed during this study are included in this article.

## Abbreviations

PVDF: Poly(vinylidene fluoride)
HRTEM: High-resolution transmission electron microscope
EC: Ethylene carbonate
EMC: Ethyl methyl carbonate
DEC: Diethyl carbonate
CV: Cyclic voltammetry
XPS: X-ray photoelectron spectroscopy
SEM: Scanning electron microscope
EDX: Energy-dispersive X-ray
OCV: Open-circuit voltage
FWHM: Full-width at half-maximum
XRD: X-ray diffraction
